# *Listeria monocytogenes* Inhibits Serotonin Transporter in Human Intestinal Caco-2 Cells

**DOI:** 10.1007/s00248-016-0809-6

**Published:** 2016-08-03

**Authors:** E. Latorre, A. Pradilla, B. Chueca, R. Pagán, E. Layunta, A. I. Alcalde, J. E. Mesonero

**Affiliations:** 1 Departamento Farmacología y Fisiología, Facultad de Veterinaria, Instituto de Investigación Sanitaria de Aragón (IIS), Universidad de Zaragoza, Zaragoza, Spain; 2 RNA-Mediated Mechanisms of Disease, Institute of Biomedical and Clinical Sciences, University of Exeter Medical School, Exeter, UK; 3 Departamento de Producción Animal y Ciencia de los Alimentos, Facultad de Veterinaria, Universidad de Zaragoza, Zaragoza, Spain; 4 Instituto Agroalimentario de Aragón – IA2, Universidad de Zaragoza – CITA, Zaragoza, Spain

**Keywords:** 5-HT, SERT, Intestinal epithelium, Listeriosis, TLR

## Abstract

*Listeria monocytogenes* is a Gram-positive bacterium that can cause a serious infection. Intestinal microorganisms have been demonstrated to contribute to intestinal physiology not only through immunological responses but also by modulating the intestinal serotonergic system. Serotonin (5-HT) is a neuromodulator that is synthesized in the intestinal epithelium and regulates the whole intestinal physiology. The serotonin transporter (SERT), located in enterocytes, controls intestinal 5-HT availability and therefore serotonin’s effects. Infections caused by *L. monocytogenes* are well described as being due to the invasion of intestinal epithelial cells; however, the effect of *L. monocytogenes* on the intestinal epithelium remains unknown. The main aim of this work, therefore, was to study the effect of *L. monocytogenes* on SERT. Caco2/TC7 cell line was used as an enterocyte-like in vitro model, and SERT functional and molecular expression assays were performed. Our results demonstrate that living *L. monocytogenes* inhibits serotonin uptake by reducing SERT expression at the brush border membrane. However, neither inactivated *L. monocytogenes* nor soluble metabolites were able to affect SERT. The results also demonstrate that *L. monocytogenes* yields TLR2 and TLR10 transcriptional changes in intestinal epithelial cells and suggest that TLR10 is potentially involved in the inhibitory effect observed on SERT. Therefore, *L. monocytogenes*, through TLR10-mediated SERT inhibition, may induce increased intestinal serotonin availability and potentially contributing to intestinal physiological changes and the initiation of the inflammatory response.

## Introduction

*Listeria monocytogenes* is a food-borne Gram-positive bacterium that might cause the serious listeriosis infection, particularly in immune-compromised individuals [1]. After the ingestion of food or contaminated water, *L. monocytogenes* crosses the intestinal barrier by invading intestinal epithelial cells. An effective immune response to *L. monocytogenes* infection relies on coordinated innate and adaptive immune responses, where the first line of defense is mediated by toll-like receptors (TLRs). However, the activation of innate immunity in response to *L. monocytogenes* infection is still not fully understood. *L. monocytogenes* has been shown to be recognized by TLR2 [2], TLR5 [3], and orphan TLR10 [4], without ruling out other immune receptors such as NOD1 [5] or NOD2 [6].

The intestinal epithelium forms a mucosal surface providing a critical barrier function against microbial invasion. Similar to immune cells, intestinal epithelial cells express TLRs and are the first line of bacterial recognition in the intestine [7]. Recent results have demonstrated that bacteria resident in the intestinal lumen, through the activation of TLRs, may affect intestinal pathophysiology [8] by acting on the intestinal serotonergic system [9]. Serotonin (5-HT) is a neuromodulator that is mainly synthesized in the intestinal epithelium, and it has been shown to regulate the whole intestinal physiology and to be essential for the maintenance of intestinal homeostasis [10–12]. In fact, the excess of extracellular 5-HT has been described as contributing to intestinal inflammation [13, 14]. 5-HT activity depends on the extracellular 5-HT availability, which is mainly modulated by the serotonin transporter (SERT) located in the enterocytes. SERT is responsible for the 5-HT uptake into these cells and controls its effects; consequently, SERT takes part in both intestinal homeostasis and inflammatory responses.

Since some studies have demonstrated that the stimulation of different TLRs modulates the intestinal serotonergic system by inhibiting SERT [15, 16], the main aim of the present work was to analyze whether *L. monocytogenes*, a pathogenic microorganism ingested with food, affects intestinal SERT activity and expression. We have used human intestinal epithelial Caco-2/TC7 cells as an in vitro model, since previous studies have shown the suitability of this cellular model to SERT and TLRs investigations [16, 17].

## Materials and Methods

### Materials

The following drugs and substances were used (abbreviations and suppliers in parentheses): serotonin (5-HT) from Sigma-Aldrich (St. Louis, MO). [^3^H]-5-HT (specific activity 28 Ci/mmol) was from PerkinElmer (Boston, MA). Goat polyclonal antibody anti-human SERT (ab130130), goat polyclonal anti-human TLR10 (ab53631), and rabbit monoclonal anti-human TLR2 (ab108998) were supplied by Abcam (Cambridge, UK). Goat polyclonal anti-human actin antibody (SC-1616r) and secondary antibodies coupled to horseradish peroxidase were from Santa Cruz Biotechnology (Santa Cruz, CA), and Pepinh-MYD (an inhibitory peptide of MyD88) was from InvivoGen (San Diego, CA). All generic reagents were purchased from Sigma-Aldrich and Roche Applied Sciences (Sant Cugat del Vallés, Barcelona, Spain).

### Cell Culture

Human enterocyte-like cell line Caco-2/TC7 [18] was maintained in high glucose DMEM supplemented with 2 mM glutamine, 100 U/ml penicillin, 100 μg/mL streptomycin, 1 % non-essential amino acids, and 20 % heat-inactivated fetal bovine serum (FBS) (from Life Technologies, Carlsbad, CA) and cultured in a humidified incubator 37 °C with 5 % CO_2_. The cell culture and management has been described elsewhere [17]. The cells were passaged enzymatically (0.25 % trypsin–1 mM EDTA) and subcultured in 25- or 75-cm^2^ plastic culture flasks and 24-well plates (Sarstedt, Germany). The medium was changed 48 h after seeding and daily thereafter. Infection experiments were carried out 14 days after seeding, and the cell medium had been free of FBS for 24 h before the experiment. Caco-2 cells were seeded in 12-well permeable polyester (PET) membranes with porous size 0.4 mm (Millicell cell culture inserts; Millipore, Billerica, MA, USA) and the growth area was 1 cm^2^. These inserts established the apical and basal compartments, and the confluence and integrity of the cells were assessed by microscopy (Zeiss Axiovert 200M with AxioCam HRC camera, Carl Zeiss Microimaging, Thornwood, NY) and a determination of transepithelial electrical resistance (TER) by epithelial voltohmmeter (Millicell Electrical Resistance System, Millipore).

### Bacterial Culture and Infection of Caco-2/TC7 Cells

During this study, cultures of *L. monocytogenes* EGD-e [19] were maintained in cryovials at −80 °C. A broth subculture was prepared by inoculating a test tube containing 5 mL of sterile tryptic soy broth (Biolife, Milan, Italy) with a colony from a plate and adding 0.6 % of yeast extract (TSBYE, Biolife). With these overnight subcultures, 250-mL Erlenmeyer flasks containing 50 mL of TSBYE were inoculated to a final concentration of 10^5^ cells/mL. These flasks were incubated under agitation (130 rpm; Selecta, mod. Rotabit, Barcelona, Spain) at 37 °C for 6 h until a cell concentration of 10^8^/mL was reached. The bacterial culture was subsequently washed with sterile PBS through two steps of centrifugation (3000×*g* for 5 min) and diluted in sterile PBS at the desired multiplicity of infection (50–200). Multiplicity of infection (MOI) is the ratio of the number of bacteria to the number of target cells.

To infect Caco-2/TC7 cells, the *L. monocytogenes* cell population was added to 0.4 mL of sterile DMEM and supplemented with 2 mM glutamine and 1 % non-essential amino acids and incubated with the Caco-2/TC7 cells at 37 °C for the periods of treatment (1 or 2 h). After that, the cell monolayer was treated with freshly prepared gentamicin solution (50 μg/mL) for 30 min to remove adherent bacteria from the cell surface. For studies about bacterial supernatants, an overnight culture supernatant was collected by centrifuging the bacterial culture and then filter-sterilized by passing it through a 0.22-μm sterile syringe filter.

For the *L. monocytogenes* growth analysis, different concentrations of serotonin (10^−4^ and 10^−8^ M) were prepared in tryptic soy broth. The tubes were then inoculated with *L. monocytogenes* (10^5^ CFU/mL) and incubated at 37 °C under agitation. The bacterial count was determined for each tube after 0, 1, 2, 3, 4, 5, 6, 7, 8, and 24 h of incubation. Briefly, 100-μL culture was obtained for each time point, serially diluted with sterile saline, and samples were pour-plated onto tryptic soy agar (Biolife) and 0.6 % of yeast extract added (TSAYE) as a recovery medium. The plates were incubated for 48 h at 30 °C. After incubation, colony-forming units (CFU) were counted with an analyzer automatic counter (Protos, Analytical Measuring System, Cambridge, UK). The limit of detection was 10 CFU/mL. Each concentration test was performed in duplicate.

### Inactivation of *L. monocytogenes*

To verify whether the inactivated *L. monocytogenes* would affect SERT, two technologies for bacterial inactivation were used: heat and pulsed electric fields (PEF) treatments. For obtaining heat-inactivated *L. monocytogenes* cells, 1 mL aliquots were immersed in a water bath at 60 °C for 1 h. The PEF treatments were carried out using equipment that delivered exponential decay pulses, as previously described [20]. The treatment chamber was made of a cylindrical plastic tube closed with two polished, stainless steel electrodes. The gap between electrodes was 0.25 cm, and the electrode area was 2.01 cm^2^. Before the treatment, *L. monocytogenes* cells were resuspended in a Mcllvaine citrate-phosphate buffer of pH 7.0, and the electrical conductivity was adjusted to 2 mS/cm for a final concentration of 10^7^ CFU/mL. Then, 0.5 mL of the samples was placed into the treatment chamber with a sterile syringe, as it has been previously described. Cell suspensions were treated for 100 pulses (1 Hz, pulse width 2 μs) at an electric field strength of 35 kV/cm, corresponding to a specific energy of 8.07 kJ/kg per pulse. In PEF experiments, the temperature of the samples was lower than 35 °C. After bacterial inactivation treatments, 0.1 mL samples were pour-plated onto TSAYE as a recovery medium. The plates were incubated for 48 h at 37 °C. The inactivation of both *L. monocytogenes* treatments (at least 5 log_10_ cell cycles) was verified before the infection of the cell culture was carried out.

### 5-HT Uptake Studies

5-HT uptake by confluent Caco-2/TC7 monolayers was previously described by our research group [21]. In brief, the cells were incubated with transport medium (137 mM NaCl, 4.7 mM KCl, 1.2 mM KH_2_PO_4_, 1.2 mM MgSO_4_, 2.5 mM CaCl_2_, 10 mM HEPES pH 7.4, 4 mM glutamine, 0.1 % BSA), and both 2 × 10^−7^ M 5-HT and 1.5 μCi/ml [^3^H]-5-HT at 37 °C for 6 min, and the uptake reaction was terminated by the twice addition of ice-cold substrate-free transport medium containing 2 × 10^−4^ M 5-HT. The cells were lysed with 0.1 N NaOH and then were analyzed for radioactivity in a liquid scintillation counter (Wallac Liquid Scintillation Counter, PerkinElmer). The total protein was measured using the Bradford method (Bio-Rad, Hercules, CA) with BSA protein as standard. The results were calculated in pmol 5-HT/mg protein and expressed as a percentage of the control value (100 %).

To analyze the involvement of TLR2 and TLR10 in the effects of *L. monocytogenes* effects on 5-HT uptake, TLR blocking was assessed. To do so, SERT activity was measured in cells pretreated with TLR2 or TLR10 antibodies at 1 μg/mL for 30 min prior to the *L. monocytogenes* infection (MOI 200) and then with the corresponding antibody for 2 h.

### Real-Time PCR Analysis

Total RNA was isolated from confluent Caco-2/TC7 cells cultured in 25-cm^2^ flaks using an RNeasy mini kit (Qiagen, Hilden, Germany) and following the manufacturer’s instructions as previously described [22]. The extracted RNA (1 μg) was used as a template for first-strand cDNA synthesis using oligo(dT) primers and a reverse transcriptase (Life Technologies). Reactions were run using the StepOnePlus Real-Time PCR System (Life Technologies) with GAPDH and HPRT1 housekeeping gene expression used as a calibrator. The primers used were HPRT1 sense (5′ CTGACCTGCTGGATTACA 3′) and HPRT1 antisense (5′ GCGACCTTGACCATCTTT 3′), GADPH sense (5′ CATGACCACAGTCCATGCCATCACT 3′) and GADPH antisense (5′ TGAGGTCCACCACCCTGTTGCTGTA 3′), TLR2 sense (5′ GAAGCTCCAGCAGGAACATC 3′) and TLR2 antisense (5′ GAATGAAGTCCCGCTTATGAAGACA 3′), TLR10 sense (5′ TTGCATTCCCACCAGGTATC 3′) and TLR10 antisense (5′ AGCCCACATTTACGCCTATC 3′), and SERT sense (5′ 5AAATCCAAGCACCCAGAGAT 3′) and SERT antisense (5′ AGACTGTGTCCCTGTGGAGA 3′). Each sample was run in triplicate, and the mean Ct was determined. The relative gene mRNA expression under each experimental condition (control or treatment) was expressed as ΔCt (= Ct_gene_ − Ct_calibrator_), and the relative gene mRNA expression was calculated as ΔΔCt (=ΔCt_control_ − ΔCt_treatment_). Finally, the relative gene expression levels were converted and expressed as a fold difference (=2^–ΔΔCt^).

### Brush Border-Enriched Fraction from Caco-2/TC7 Cells and Western Blotting

The enriched fraction of the intestinal brush border was prepared following the procedure previously described [23]. The brush border-enriched fraction and the cell lysate (60 μg of total protein) from Caco-2/TC7 cells were electrophoresed in 8 % SDS-PAGE gels and then transferred to PVDF membranes by electroblotting. The membranes were blocked with 5 % nonfat dried milk plus 1 % BSA and probed using a specific primary antibody (anti-SERT 1:500, anti-TLR2 1:1000, and anti-TLR10 1:1000). The primary antibody was detected using a secondary antibody coupled to horseradish peroxidase and the WesternBright Sirius Kit (Advansta, Menio Park, CA) and was visualized with VersaDoc (Imaging System, Bio-Rad). The blots were reprobed after stripping, with goat polyclonal anti-human β-actin used to determine differences in the sample load. The specific protein/β-actin protein ratio was calculated in densitometric units from the film with Quantity One Analysis Software (Bio-Rad), and the results were expressed as a percentage of the control values.

### Statistical Analysis

All results are expressed as mean ± the standard error of the mean (SEM). Statistical comparisons were performed by one-way ANOVA followed by the Bonferroni posttest with a confidence interval of 95 % (*P* < 0.05). The normal distribution has been previously confirmed with the D’Agostino-Pearson test. The statistical analysis was carried out with the computer-assisted GraphPad Prism Program (Prism version 4.0, GraphPad Software, San Diego, CA).

## Results

### *L. monocytogenes* Inhibits 5-HT Uptake

**Fig. 1:**
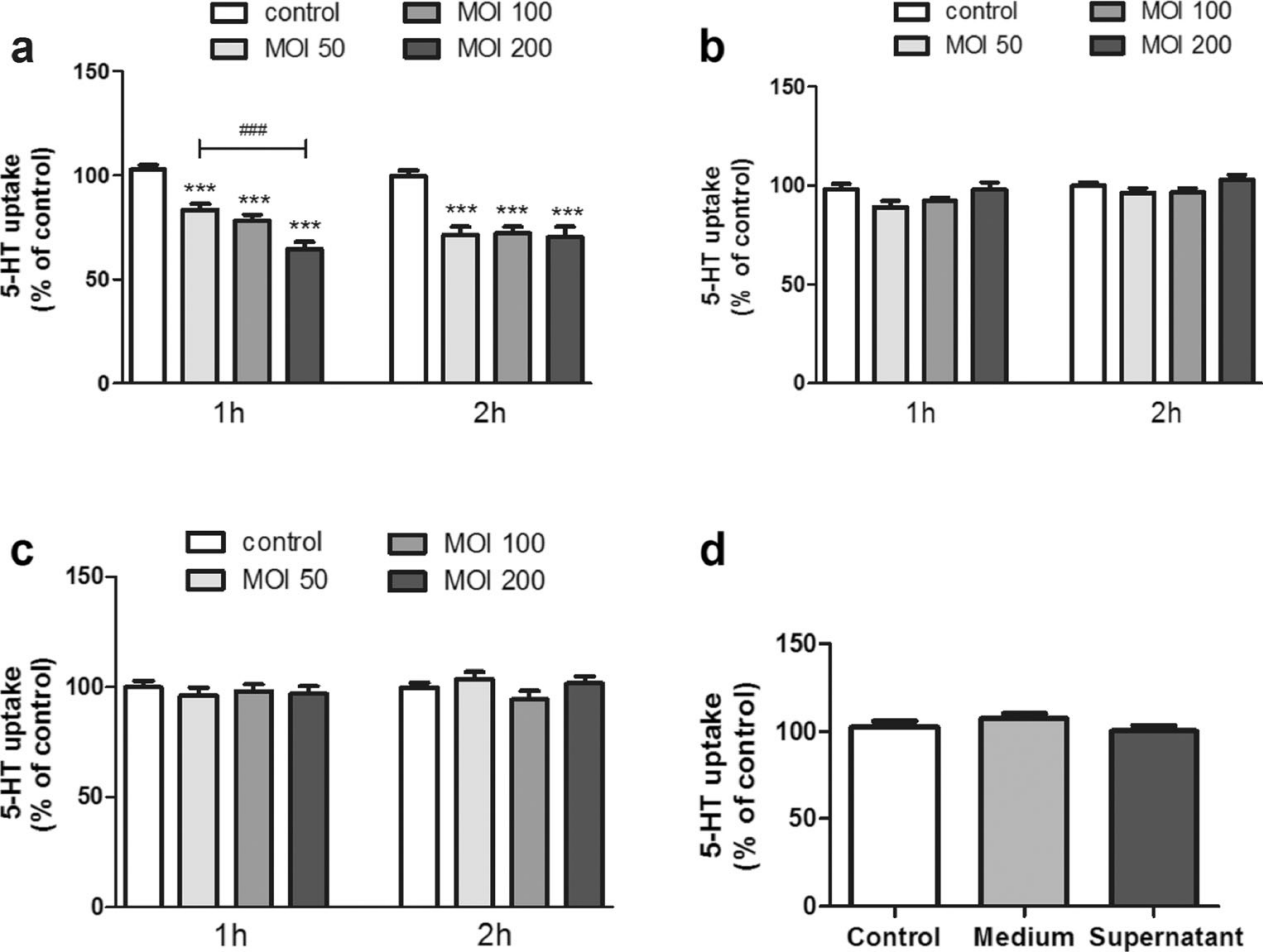
*L. monocytogenes* effects on 5-HT uptake. Cells were infected with alive or inactivated *L. monocytogenes* at MOI 50, 100, and 200 for 1 or 2 h. 5-HT uptake was measured after 6 min incubation with 0.2 μM 5-HT. Control condition corresponds to untreated cells. **a** Effect of live *L. monocytogenes* on 5-HT uptake. The results are expressed as the percentage of the uptake control and are the mean ± SE of six biological replicates in seven independent experiments (*n* = 42). ***P < 0.001 compared with the control. **b** Effect of heat-killed *L. monocytogenes* on 5-HT uptake. The results are expressed as the percentage of the uptake control and are the mean ± SE of six biological replicates in seven independent experiments (*n* = 42). **c** Effect of *L. monocytogenes* inactivated by PEF on 5-HT uptake. The results are expressed as the percentage of the uptake control and are the mean ± SE of three biological replicates in four independent experiments (*n* = 12). **d** Effect of *L. monocytogenes* supernatant on 5-HT uptake. The results are expressed as the percentage of the uptake control and are the mean ± SE of three biological replicates in four independent experiments (*n* = 12)

To determine if *L. monocytogenes* affects 5-HT uptake, the Caco-2/TC7 monolayer was first studied; the confluence and integrity of the cells after *L. monocytogenes* infection were assessed by microscopy and by determining the TER. The infection of *L. monocytogenes* at the maximum concentration used in this study (MOI 200 or 200 bacteria per cell) for 2 h did not affect neither the morphology nor the TER of the cell monolayer (data not shown). Next, we examined the effect of *L. monocytogenes* on the 5-HT uptake for 1 and 2 h at different MOIs (50, 100, and 200). The results showed that *L. Monocytogenes* causes a significant inhibition of 5-HT uptake and that this inhibition is bacterial load-dependent for the 1-h infection; however, for the 2-h infection, the same reduction was observed at different MOIs (Fig. 1a).

To characterize the SERT inhibition by *L. monocytogenes*, we examined the effect of treated Caco-2/TC7 monolayers with inactivated *L. monocytogenes*. Initially, *L. monocytogenes* was inactivated by heat; however, the results obtained showed that this was not able to affect SERT activity (Fig. 1b).

Extending this result, heat could damage to most microbial structures and components, limiting their effects on SERT activity. Thus, a different inactivation technology that is less harmful to microbial structures was used. To this end, PEF treatment, a less aggressive method that is known to only affect cell envelopes, was used [20]. The results showed that *L. monocytogenes* inactivated by PEF was not able to affect SERT (Fig. 1c).

We also examined the effect of *L. monocytogenes* culture supernatant to evaluate its ability to produce toxins that could affect 5-HT uptake. The results showed that no effect of *L. monocytogenes* supernatant on 5-HT uptake, indicating that only living *L. monocytogenes* cells seem to affect SERT activity (Fig. 1d).

### The Effects of *L. monocytogenes* on SERT Molecular Expression

**Fig. 2:**
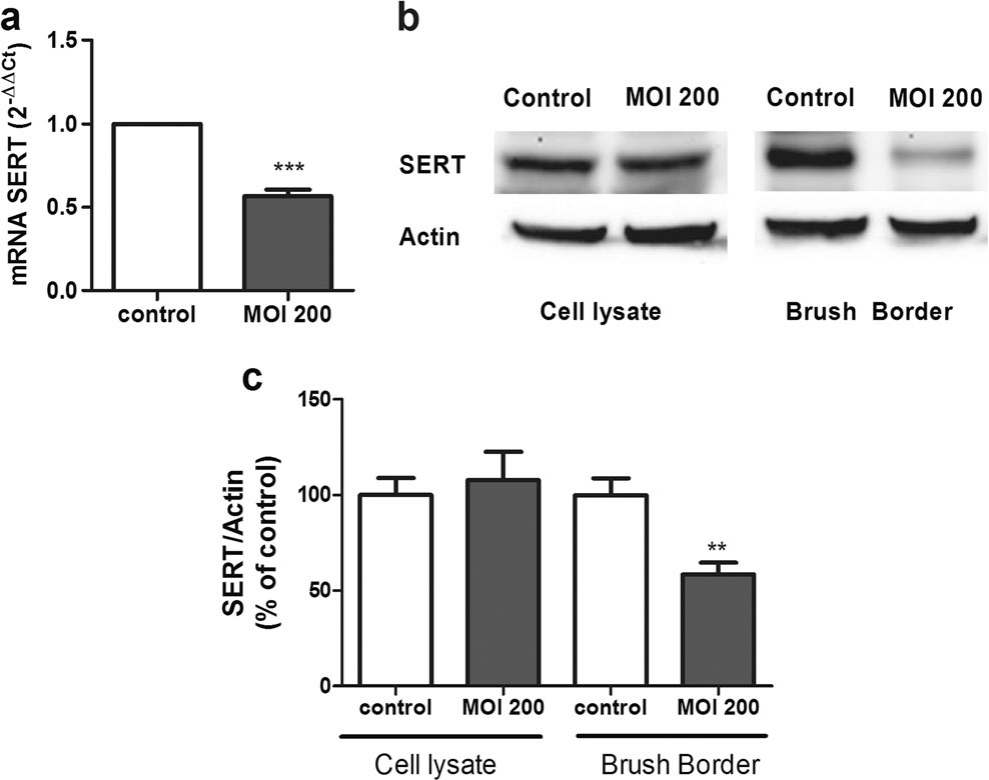
*L. monocytogenes* inhibit SERT mRNA and protein expression. **a** Quantitative RT-PCR analysis of SERT mRNA expression in cells infected with *L. monocytogenes* for 2 h at MOI 200. Relative quantification was performed using comparative Ct (2^−ΔΔCt^) of three biological replicates in five independent experiments (*n* = 15). Results are expressed as arbitrary units of control = 1. ***P < 0.001 compared with the control value. **b** Immunodetection of SERT by western blot in cell lysate and brush border from Caco-2/TC7 cells infected with *L. monocytogenes* for 2 h at MOI 200. **c** Quantification of SERT protein in both cell lysate and brush border using β-actin as an internal control of the protein load (SERT/β-actin ratio). The results are expressed as a percentage of the control value and are the mean ± SEM of two biological replicates in five independent experiments (*n* = 10). **P < 0.01 compared with the control value

To analyze the effects of *L. monocytogenes* on SERT activity in more depth, SERT molecular expression was assessed. Thus, SERT mRNA and protein levels were measured in Caco-2/TC7 cells that had been treated for 2 h with MOI 200. The results showed that *L. monocytogenes* infection decreases SERT mRNA level (Fig. 2a). In relation to SERT protein analysis, the results showed that SERT was significantly diminished in the brush border of cells infected with *L. monocytogenes*; however, the SERT protein level in the whole cell homogenate was not modified (Fig. 2b, c).

### The Role of MyD88 on *L. monocytogenes* Effects on SERT Function

**Fig. 3:**
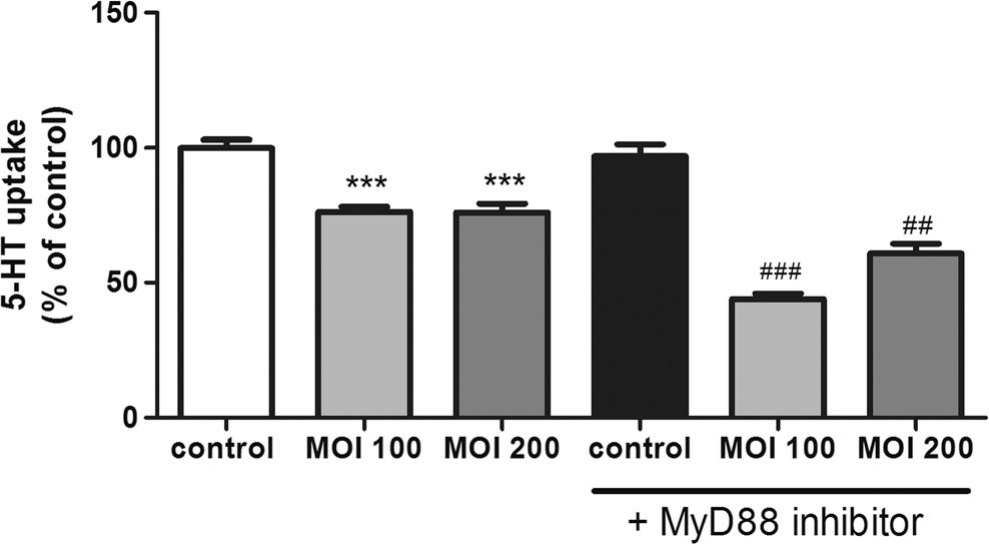
MyD88 involvement on *L. monocytogenes* effect on 5-HT uptake. Cells were infected with *L. monocytogenes* at MOI 100 and 200 for 2 h and/or MyD88 inhibitor (100 μM 1 h previous and during the infection with *Listeria*) and compared with untreated cells (control). Uptake of 5-HT was measured after 6 min of incubation, and 5-HT concentration was 0.2 μM. The results are expressed as the percentage of the uptake control and are the mean ± SE of three biological replicates in four independent experiments (*n* = 12). ***P < 0.001 compared with the control. ^###^ *P* < 0.001 and ^##^ *P* < 0.01 compared with the corresponding MOI effect without MyD88 inhibitor

TLRs have been clearly established as the major sensors of the innate immune system, and they seem to play a key role in immunity against *L. monocytogenes* infection. MyD88 is a universal adapter protein used by most of TLRs to start their effects. Thus, to clarify the effects of *L. monocytogenes* on SERT, the role of MyD88 was analyzed. SERT activity was measured in cells treated with Pepinh-MYD (an inhibitory peptide of MyD88) plus a *L. monocytogenes* infection. The results showed that MyD88 inhibition did not alter 5-HT uptake in control conditions; however, MyD88 blocking significantly increased the inhibitory effect of *L. monocytogenes* on 5-HT uptake (Fig. 3).

### Involvement of TLR2 and TLR10 in the Effects of *L. monocytogenes* on SERT

**Fig. 4:**
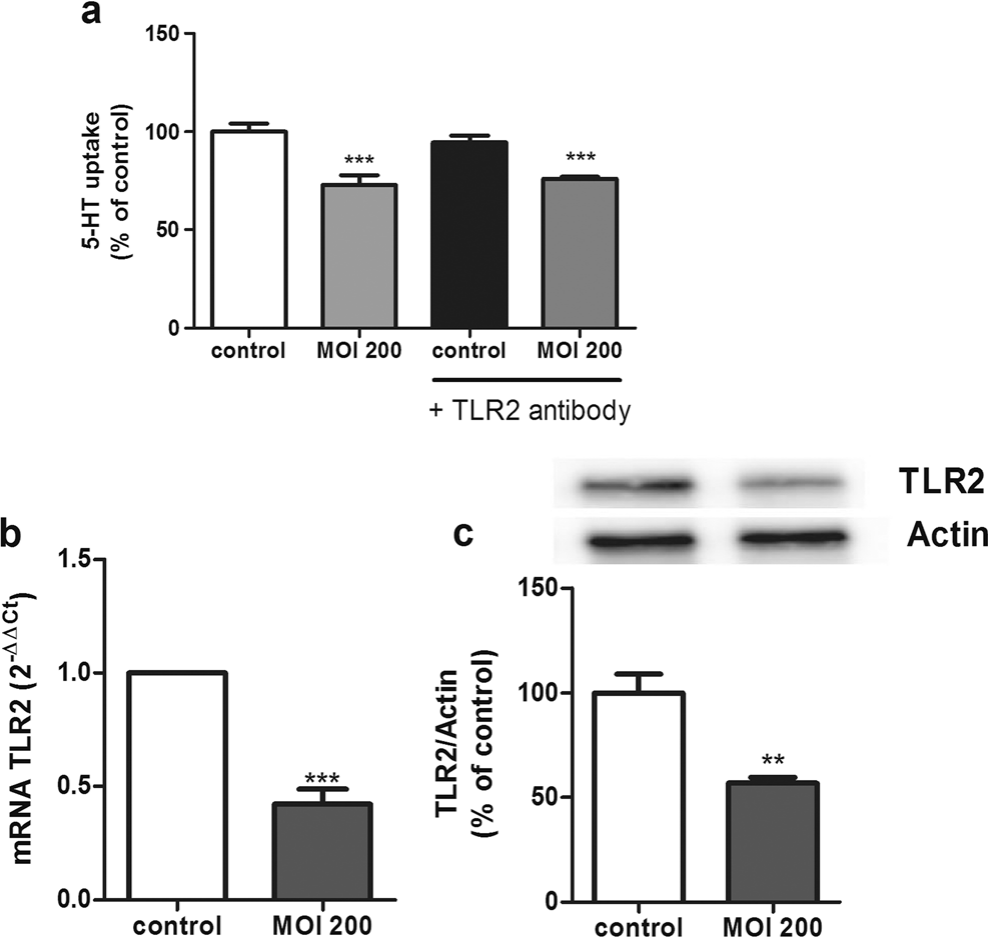
TLR2 involvement on *L. monocytogenes* effects. **a** Cells were infected with *L. monocytogenes* at MOI 200 for 2 h and/or TLR2 antibody (1 μg 30 min previous the infection) and compared with untreated cells (control). Uptake of 5-HT was measured after 6 min of incubation, and 5-HT concentration was 0.2 μM. The results are expressed as the percentage of the uptake control and are the mean ± SE of three biological replicates in four independent experiments (*n* = 12). ***P < 0.001 compared with the control. **b** Quantitative RT-PCR analysis of TLR2 mRNA expression in cells infected with *L. monocytogenes* for 2 h at MOI 200. Relative quantification was performed using comparative Ct (2^−ΔΔCt^) of three biological replicates in five independent experiments (*n* = 15). Results are expressed as arbitrary units of control = 1. ***P < 0.001 compared with the control value. **c** Expression and quantification of TLR2 protein levels in cell lysate using β-actin as an internal control of the protein load (TLR2/β-actin ratio). The results are expressed as a percentage of the control value and are the mean ± SEM of two biological replicates in four independent experiments (*n* = 8). **P < 0.01 compared with the control value

As recent studies have postulated that both TLR2 and TLR10 play a key role in the inflammatory response against *L. monocytogene*s [4] and in order to clarify the previous results, the involvement of TLR2 and TLR10 was studied. TLR’s role was analyzed by blocking TLR2 or TLR10 with specific antibodies. To do so, SERT activity was measured in cells pretreated with the TLR2 antibody at 1 μg/mL for 30 min prior to a *L. monocytogenes* infection (MOI 200) along with the TLR2 antibody for 2 h. The results showed that the TLR2 blocking did not modify the inhibitory effect of *L. monocytogenes* on 5-HT uptake, so TLR2 does not seem to be involved in the effects of *L. monocytogenes* on SERT activity (Fig. 4a). Interestingly, the *L. monocytogenes* infection affected TLR2 expression by decreasing both TLR2 mRNA and protein (Fig. 4b, c).

**Fig. 5:**
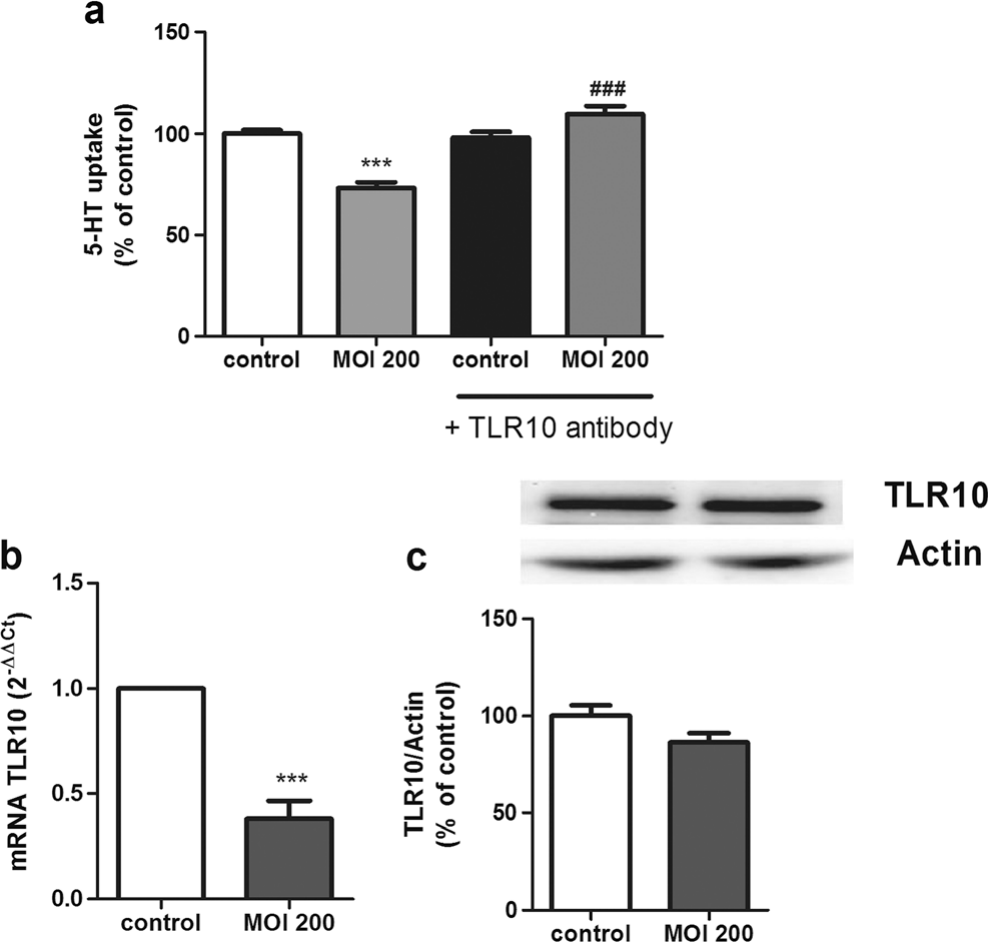
TLR10 involvement on *L. monocytogenes* effects. **a** Cells were infected with *L. monocytogenes* at MOI 200 for 2 h and/or TLR10 antibody (1 μg 30 min previous the infection) and compared with untreated cells (control). Uptake of 5-HT was measured after 6 min of incubation, and 5-HT concentration was 0.2 μM. The results are expressed as the percentage of the uptake control and are the mean ± SE of three biological replicates in four independent experiments (*n* = 12). ***P < 0.001 compared with the control. ^###^ *P* < 0.001 compared with MOI 200 effect without antibody. **b** Quantitative RT-PCR analysis of TLR10 mRNA expression in cells infected with *L. monocytogenes* for 2 h at MOI 200. Relative quantification was performed using comparative Ct (2^−ΔΔCt^) of three biological replicates in five independent experiments (*n* = 15). Results are expressed as arbitrary units of control = 1. ***P < 0.001 compared with the control value. **c** Expression and quantification of TLR10 protein in cell lysate using β-actin as an internal control of the protein load (TLR10/β-actin ratio). The results are expressed as a percentage of the control value and are the mean ± SEM of two biological replicates in four independent experiments (*n* = 8)

Regarding TLR10, we first described TLR10 expression in our cell model by analyzing mRNA and protein expression (data not shown), as the expression of TLR10 was unknown in Caco-2/TC7 cells. Then, TLR10 involvement was analyzed by blocking TLR10 with a specific antibody (1 μg/mL for 30 min before the infection with *L. monocytogenes* MOI 200 along with the TLR10 antibody for 2 h), and 5-HT uptake was analyzed. The results showed that the effect of *L. monocytogenes* on SERT was reversed by blocking TLR10, suggesting that this TLR could mediate the effects of *L. monocytogenes* on SERT activity (Fig. 5a). Moreover, Caco-2/TC7 cells infected with *L. monocytogenes* seemed to present an altered TLR10 expression. Indeed, the results showed that *L. monocytogenes* causes a decrease in the TLR10 mRNA level; however, the TLR10 protein level did not seem to be modified (Fig. 5b, c), possibly due to the short time of infection.

### The Effects of 5-HT on *L. monocytogenes* Growth

**Fig. 6:**
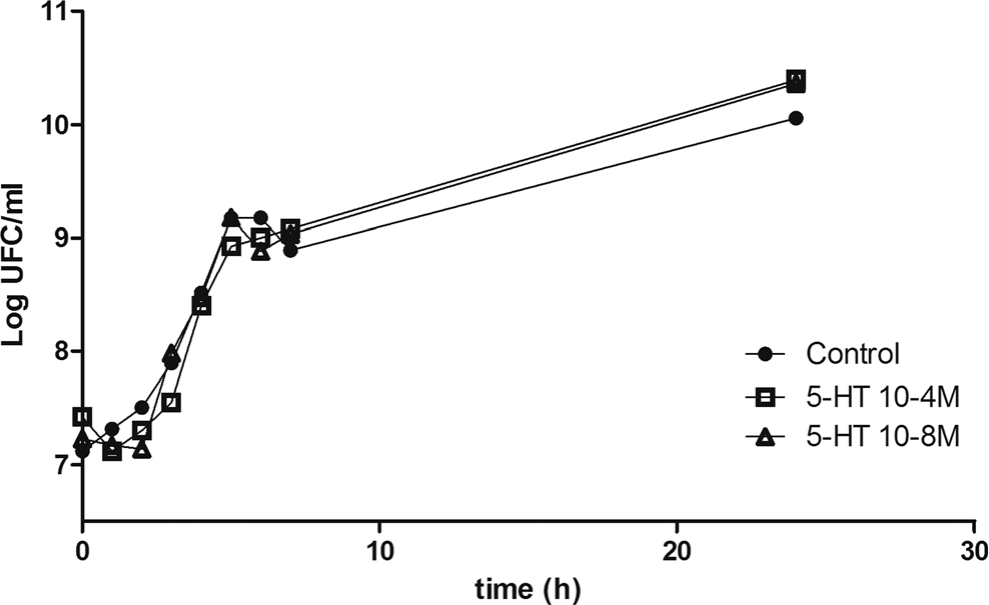
5-HT effect on growth curve of *L. monocytogenes. L. monocytogenes* growth was evaluated adding increasing concentrations of 5-HT 10^−8^ M and 10^−4^ M to the bacterial medium from 0 to 24 h. Results are expressed as the mean of three independent experiments in duplicate

Our results demonstrate that *L. monocytogenes* inhibits SERT. Consequently, this effect may trigger an increase of extracellular 5-HT availability in the intestinal lumen, which in turn may yield possible feedback on *L. monocytogenes*. To study this hypothesis, *L. monocytogenes* growth was evaluated by adding increasing concentrations of 5-HT to the bacterial medium. As a physiological condition, 5-HT 10^−8^ M was used, and as an inflammatory condition, 5-HT 10^−4^ M was used. The results showed that 5-HT (at any concentration assayed) did not seem to modify *L. monocytogenes* growth (Fig. 6).

## Discussion

Understanding host-microbe interaction in the intestinal tract may provide new insights into intestinal homeostasis and inflammation. Recent studies have demonstrated that some gut microbes can modulate serotonergic system expression, promote 5-HT biosynthesis from enterochromaffin cells, and directly produce or release serotonin [24, 25], consequently contributing to intestinal homeostasis. Moreover, some TLRs have also been reported to regulate SERT activity and expression [15, 16]. Therefore, our main aims in this study were to analyze the effects of *L. monocytogenes* on intestinal SERT activity and expression and to determine the TLRs involved.

Our results showed that living *L. monocytogenes* cells cause a significant and MOI-dependent reduction of 5-HT uptake for 1 h of infection, with the maximum effect occurring at MOI 200. The inhibitory effect of *L. monocytogenes* on intestinal 5-HT uptake did appear to require direct contact between living bacteria and the intestinal epithelial cells, as inactivated *L. monocytogenes* cells and bacterial supernatants did not modify 5-HT uptake. Several studies have shown that entry of *L. monocytogenes* into the cells is dependent on cell polarization and differentiation [26, 27]. In fact, *L. monocytogenes* is able to invade differentiated polarized Caco-2 cells predominantly through the basolateral surface [26]. Our study was carried out in 14-day-old Caco-2 monolayers, when the cells have reached total confluency and developed enterocyte differentiation [18]. At this culture stage, no basolateral surfaces are accessible to bacteria, and levels of invasion dramatically decrease compared with invasion of non-confluent monolayers [26]. In addition, our results showed not TER change, indicating that the integrity of the cell monolayer was undamaged. Then, the observed effect on SERT could be a preliminary result of *L. monocytogenes* prior to intestinal epithelial cells invasion. Nevertheless, we cannot completely discard a possible *L. monocytogenes* cell invasion.

Our results agree with previous studies that demonstrated the inhibitory effects of living *L. monocytogenes* on the serotonergic system in the vascular system [27, 28]. However, a recent study has described effects of heat-inactivated *L. monocytogenes* on the immune response of human monocytic THP-1 cells [29].

The inhibitory effect of *L. monocytogenes* seems to be due to a decrease of SERT protein expression in the brush border cell membrane, which is probably due to posttranslational or posttranscriptional mechanisms. *L. monocytogenes* yielded a reduction of SERT mRNA; however, only SERT protein expression in the brush border was affected; this could be due to the processing time, as the bacteria are only in contact with the cells for 2 h, so they may need more time to reduce SERT total protein. These results suggest that *L. monocytogenes* may inhibit SERT activity by reducing SERT protein expression in the brush border for the short term and that, in the long term, this bacterium might reduce SERT protein levels in cell lysate, since it reduces SERT mRNA level. In agreement with these results, other pathogenic bacteria, like *E. coli*, have also been shown to decrease intestinal SERT activity and expression [30].

SERT activity can also be reduced by 5-HT [22], and some microbes are known to directly produce 5-HT by themselves [31], so *L. monocytogenes* could affect SERT in this way; however, *L. monocytogenes* has not been described as a 5-HT producer.

Regarding the role of MyD88, the results surprisingly showed that MyD88 blocking increases the inhibitory effects of *L. monocytogenes*, so it seems that *L. monocytogenes* may activate both a MyD88-dependent (which may increase 5-HT uptake) and a MyD88-independent pathway (which may decrease 5-HT uptake). These results, like other studies, support the hypothesis that *L. monocytogenes* can activate different pathways, both dependent on and independent of MyD88 [32].

TLRs have been clearly established as the major sensors of the innate immune system, and they seem to play a key role in immunity against *L. monocytogenes* infection. Although TLR2 [33] and MyD88 [34] have been shown to detect *L. monocytogenes* infections in other cell types, only NOD2 has been directly shown to mediate the inflammatory response to *L. monocytogenes* in the intestine [6]. Our investigation of the role of TLRs in *L. monocytogenes* infection has yielded several observations. TLR2 alone did not seem to play a role in 5-HT uptake in response to *L. monocytogenes*, but intestinal epithelial cells have shown a TLR2 expression inhibition by *L. monocytogenes*. Our results disagree with previous results that described a possible relationship between TLR2 and *L. monocytogenes*, although these studies were conducted in immune cells [35, 36].

In this study, the results indicated that TLR10 may play a role in the effects of *L. monocytogenes* on intestinal SERT activity. The highest level of TLR10 expression has been reported in immune cells [37, 38], but TLR10 mRNA and protein expression have previously been shown in human intestinal epithelial cell lines, such as SW480 and HT-29 [4, 39]. Polarized expression of TLRs is a common mechanism for preventing unnecessary and potentially detrimental inflammatory response to bacteria. Differential TLR expression is observed in intestinal epithelial cells depending on the local commensal flora. Generally, TLR1 to TLR10 has been reported on the cell apical side, but only a low constitutive expression of TLR2, TLR4, and TLR5 has been observed on the cell basolateral side [40]. Our study has demonstrated TLR10 expression in the Caco-2/TC7 cell line, indicating a possible functional role in the intestinal epithelium. Although the TLR10 signaling pathway remains unknown, TLR10 expression is located in the cell membrane and has a very strong structural association with TLR1 and TLR6, both of which are heterodimeric with TLR2 [41]. Some studies have suggested a possible TLR2/TLR10 dimer in response to *L. monocytogenes* [4, 42], but this hypothesis disagrees with our results, as TLR10 blocking is enough to reverse the effect of *L. monocytogenes* on SERT activity. However, supporting our results, a recent study has suggested a possible TLR10/TLR10 dimer [42].

TLRs have been described as being activated by pathogen-associated molecular patterns (PAMPs). Each TLR has different PAMPs to be activated, for instance TLR4 detects LPS from Gram-negative bacteria and TLR2 recognizes lipoteichoic acid from Gram-positive bacteria. However, TLR10 agonist is still unknown. Our results suggest that TLR10 could be activated in a different way, as only living *L. monocytogenes* seems to have effects on SERT. A recent hypothesis has indicated that some TLRs can detect microbial viability using vita-PAMPs, a special class of PAMPs that recognize microbial life [43, 44]; thus, TLR10 may be a receptor for microbial viability and the TLR10 ligand could be a vita-PAMP. In support of this hypothesis, recent studies have demonstrated TLR10’s effects only with living bacteria [4, 45].

Regarding a possible feedback, our results demonstrated that 5-HT does not alter *L. monocytogenes* growth. These results are in agreement with recent studies, which have described that 5-HT does not modify microbial growth; however, 5-HT could alter microbial virulence behavior and change the composition of the intestinal microbiota [46, 47].

In summary, our study demonstrates for the first time that only living *L. monocytogenes* cells lead to a significant reduction in 5-HT uptake in human intestinal epithelial cells and that this inhibitory effect can be mediated through TLR10. The present work contributes to the understanding of intestinal serotonergic responses induced by gut microbes.
